# Supplementary material for: Development of a computer-aided design software for the quantitative evaluation of aesthetic damage
Source: PLoS One. 2019 Dec 18;14(12):e0226322. doi: 10.1371/journal.pone.0226322 (PMC6919621; doi:10.1371/journal.pone.0226322)
Supplement: S1 Code site — 1. (ZIP) [file pone.0226322.s001.zip › [EESC_jr][Projeto]Relatorio_07_21/reports/templates_zip/medialoot-lumino-admin-bootstrap-template.zip!/Lumino Admin Bootstrap Template/MediaLoot_License_Agreement.pdf]

MediaLoot License Agreement

By subscribing to MediaLoot, purchasing an individual resource ("item" or "file"), or downloading a free resource you are being granted a license to use these files for specific uses under certain conditions. Ownership remains with MediaLoot and the respective copyright holders, and you are required to abide by the following licensing terms.

-----FREE LICENSE----
If you have downloaded this file without paying for an individual use license or a MediaLoot subscription, you are goverened by the free license. The free license grants you the exclusive right to use and incorporate these files in your personal or commercial projects under the following conditions:

1. All uses of these free items must contain an attribution link back to MediaLoot.com or the specific download page of this resource.

2. You may use this resource or adaptations of this resource in commercial or other projects for single-application purposes. For example, using this work inside of a client project is acceptable, but using it on a tee-shirt design that will be distributed to multiple people is not.

3. You MAY NOT distribute or resell this resource, or its derivatives, and you may not upload this resource to other websites. All sharing of this resource must be done using a link to the MediaLoot download page.

----REGULAR LICENSE----
If you have paid for an individual use license or were paying for a MediaLoot subscription at the time of download, you are governed by the regular license. The regular license grants you, the purchaser, the exclusive and non-transferrable rights to use this resource under the following conditions:

1. You may use this resource or adaptations of this resource in any personal or commercial single-use applications. This includes print applications, online or digital applications, and any other application where the resource is incorporated or used for one individual project or use.

2. You are not required to attribute or link to MediaLoot in any of the acceptable use conditions.

3. You may include and use this resource in projects or applications that will be sold to a single individual for a single use. For example, you may include this resource in a website you will be designing for a client.

4. You MAY NOT individually resell or distribute this resource, and you may not upload this resource to other websites. Additionally, you may not include this resource in other projects that will be distributed or subjected to multiple-use applications.

5. You MAY NOT use this resource or its derivatives in multi-use applications. This includes distribution, resale, or resale of any products that contain this resource. Any application that will distribute or use this file for multiple applications is governed by the Extended License.

----EXTENDED LICENSE----

MediaLoot is not currently granting extended use licenses or any licenses for distribution, resale, or multi-use applications. If you are interested in purchasing a license of this nature, please contact info@medialoot.com
